# Intra-operative adherence to lung-protective ventilation: a prospective observational study

**DOI:** 10.1186/s13741-016-0033-4

**Published:** 2016-04-27

**Authors:** Jaimin M. Patel, Roisin Baker, Joyce Yeung, Charlotte Small, On behalf of West Midlands-Trainee Research and Audit Network (WM-TRAIN)

**Affiliations:** Institute of Inflammation and Ageing, College of Medical and Dental Sciences, University of Birmingham, Birmingham, UK; Department of Anaesthesia, University Hospital Birmingham NHS Foundation Trust, Birmingham, UK

## Abstract

**Background:**

Lung-protective ventilation in patients with acute respiratory distress syndrome improves mortality. Adopting this strategy in the perioperative period has been shown to reduce lung inflammation and postoperative pulmonary and non-pulmonary sepsis complications in patients undergoing major abdominal surgery. We conducted a prospective observational study into the intra-operative ventilation practice across the West Midlands to assess the use of lung-protective ventilation.

**Methods:**

Data was collected from all adult ventilated patients undergoing surgery across 14 hospital trusts in the West Midlands over a 2-day period in November 2013. Data collected included surgical specialty, patient’s biometric data, duration of procedure, grade of anesthetist, and ventilatory parameters. Lung-protective ventilation was defined as the delivery of a tidal volume between 6 and 8 ml/kg/predicted body weight, a peak pressure of less than 30 cmH_2_O, and the use of positive end expiratory pressure of 6–8 cmH_2_O. Categorical data are presented descriptively, while non-parametric data are displayed as medians with statistical tests from Mann-Whitney *U* tests or Kruskal-Wallis tests for independent samples while paired samples are represented by Wilcoxon signed rank tests.

**Results:**

Four hundred six patients with a median age of 56 years (16–91) were included. The majority of operations (78 %) were elective procedures with the principal anesthetist being a consultant. The commonest surgical specialties were general (29 %), trauma and orthopedic (19 %), and ENT (17 %). Volume-controlled ventilation was the preferred ventilation strategy in 70 % of cases. No patients were ventilated using lung-protective ventilation. Overall peak airway pressure (pPeak) was low (median 20 cmH_2_O (inter-quartile range [IQR] 10–43 cmH_2_O)) with median delivered tidal volumes of 8.4 ml/kg/predicted body weight (PBW) (IQR 3.5–14.5 ml/kg/PBW). The median positive end expiratory pressure (PEEP) was only 4 cmH_2_O (0–5 cmH_2_O) with PEEP not used in 152 cases.

**Conclusions:**

Perioperative lung protection ventilation can improve patient outcomes from major surgery. This large prospective study demonstrates that within the West Midlands lung-protective ventilation during the perioperative period is uncommon, especially in relation to the use of PEEP, and that perhaps further trials are required to promote wider adoption of practice.

## Background

The concept of lung-protective ventilation is now well established amongst critically ill patients and is a standard care for ventilated patients in intensive care units (ICUs) who have features of acute lung injury and also in those who do not. Lung-protective ventilation employs low tidal volume ventilation (6 ml/kg/predicted body weight) with plateau pressure of 30 cmH_2_O or less (Ventilation with Lower Tidal Volumes as Compared with Traditional Tidal Volumes for Acute Lung Injury and the Acute Respiratory Distress Syndrome, 2000; Amato et al. 1998). There is overwhelming evidence that employing such a strategy significantly reduces mortality from acute respiratory distress syndrome (ARDS), increases the number of ventilator-free days, and reduces the need for organ support in the ICU (Ventilation with Lower Tidal Volumes as Compared with Traditional Tidal Volumes for Acute Lung Injury and the Acute Respiratory Distress Syndrome, 2000; Amato et al. 1998; Barbas et al. 2005; Sakr et al. 2005).

Although an established concept in ICUs, the optimal ventilator strategies to employ within the perioperative period remain uncertain. There is emerging evidence that even short episodes of invasive ventilation, such as those undertaken in the perioperative period, can cause ventilator-induced lung injury (VILI) by barotrauma, volutrauma, and atelectrauma, as well as causing a pro-inflammatory response evidenced by an increase in pulmonary tumor necrosis factor-alpha (TNF-α) and interleukin-6 (IL-6) (Barbas et al. 2005; Slutsky & Ranieri 2013; Imai et al. 2003; Lim & Wagner 2003; Tremblay et al. 2002). The potential injury caused to the lungs by high-volume, high-pressure ventilation in conjunction with the systemic inflammatory response (SIRS) to surgery have led researchers to postulate these as mechanisms that contribute to pulmonary and non-pulmonary organ dysfunction in patients undergoing surgery (Lellouche et al. 2012; Wrigge et al. 2004).

Postoperative pulmonary complications (PPCs) have been a particular focus as they are the second commonest complication after surgical site infections and are the major cause of morbidity and mortality. It is estimated that the risk of PPC is between 2 and 19 % (Fisher et al. 2002). Risk factors for PPCs include upper abdominal surgery, obesity, chronic lung disease, the duration of anesthesia, the presence of nasogastric tubes postoperatively, and emergent surgery (Fisher et al. 2002; Guldner et al. 2015; Serejo et al. 2007).

However, the translation of these biological and physiological changes to clinically relevant outcomes remain contentious with randomized controlled trials (RCTs) reporting conflicting results. The publication of Futier et al.’s trial in 2013 attempted to address the issue of perioperative ventilation. In this large RCT, the use of a protective ventilation strategy (including recruitment maneuvers) was compared to standard ventilation in patients undergoing major abdominal surgery. They demonstrated that use of a lung-protective strategy significantly reduced the incidence of pulmonary and non-pulmonary sepsis (Futier et al. 2013).

Evidence for a lung-protective strategy intra-operatively is growing; however, the exact mechanism of how this should be delivered remains uncertain, with levels of positive end expiratory pressure (PEEP) and the use of recruitment maneuvers, tidal volumes, and peak pressures still being debated (Guldner et al. 2015; Anaesthesiology PNIftCTNotESo et al. 2014; Severgnini et al. 2013; Tao et al. 2014).

The aim of this study was to assess the ventilator strategies being employed amongst ventilated patients undergoing surgery within the West Midlands region of the UK and to ascertain whether anesthetists employ a perioperative lung-protective strategy.

## Methods

This was a prospectively designed study of ventilation practices over a 2-day period between the 1^st^ and 2^nd^ of November 2013 within 14 out of the 17 acute hospital trusts within the West Midlands Deanery region. The West Midlands is a large region in the heart of the UK, and the trusts involved included both district general hospitals and large tertiary centers.

The study did not require ethical approval or research registration as anonymous observational data was collected and patients’ outcomes were not investigated. This was confirmed by the online NRES decision tool (http://www.hra-decisiontools.org.uk/research/) and the research and development departments at the University of Birmingham. Each participating site had the study registered as a clinical audit in their departments with appropriate oversight conducted by principal investigators (consultant anesthetist). Patient consent was not deemed necessary as this study was focusing on the ventilation practices of anesthetists and no outcome data was collected.

Independent anesthetists not involved with the delivery of anesthesia collected data. Data was collected prospectively during anesthesia to include demographic data (age, gender, and American Society of Anesthesiology [ASA]) and biostatical data (height and weight) to allow derivation of predicted body weight (PBW), as previously described by the ARDS Network (Ventilation with Lower Tidal Volumes as Compared with Traditional Tidal Volumes for Acute Lung Injury and the Acute Respiratory Distress Syndrome, 2000). Operative details collected included type of surgery, duration of surgery, the nature of surgery (emergency/elective), and the grade of the principal anesthetist. Details regarding the mode of ventilation, the tidal volumes being delivered, PEEP, peak pressure (pPeak), and percentage of inspired oxygen (FiO_2_) were collected in real time from the anesthetic machines. The delivery of recruitment maneuvers was collected by asking the principal anesthetist if any had been performed during the course of the procedure and, if so, how many and at what time intervals.

All patients undergoing surgery who were intubated with an endotracheal tube were eligible. Patients undergoing thoracic surgery (including esophagectomy) and cardiopulmonary bypass surgery were excluded. The ARISCAT group demonstrated that patients undergoing intra-abdominal surgery were at high risk of developing PPCs, while patients undergoing orthopedic, gynecological, and ENT procedures had about a 2 % risk of developing respiratory complications postoperatively. Based on this, and the definition of high-risk surgery from the IMPROVE study, a pre-defined subgroup analysis of patients undergoing major abdominal surgery (greater than 2 h) was performed to reflect a population at high risk of developing PPCs (Futier et al. 2013).

Lung-protective ventilation was defined according to a modified strategy employed by Futier et al. in the IMPROVE trial. They used a delivered tidal volume of 6–8 ml/kg/PBW, PEEP of 6–8 cmH_2_O, a plateau pressure (pPlateau) of less than 30 cmH_2_O, the use of recruitment maneuvers (30 cmH_2_O for 30 s) every 30 min, and a FiO_2_ of less than 50 %. As the use of recruitment maneuvers is not routine practice and remains highly contentious with some authors reporting significant cardiovascular instability, the use of recruitment maneuvers was excluded from the definition of lung protection for the purposes of this study (Anaesthesiology PNIftCTNotESo et al. 2014; Nielsen et al. 2005; Minkovich et al. 2007).

The National Audit Project (NAP) 4 estimated that each year 2.9 million patients undergo general anesthesia within the UK and that in 35 % of cases an endotracheal tube is utilized (1 million patients). Based on these figures, it was estimated that 7900 operations occur daily across the UK and that within the 14 centers partaking in this study we would anticipate 570 general anesthetics a day, of which 200 (35 %) would fulfill the inclusion criteria. Therefore, it was predicted that approximately 400 cases would be collected within the 2 days.

Categorical data are displayed as total number (%). Continuous data were tested for normality using a Shapiro-Wilk test and demonstrated the data to be non-parametric. Therefore, data are displayed as medians (inter-quartile range, IQR) and a Wilcoxon signed rank test used to compare paired samples. Where more than two groups have been analyzed, a Kruskal-Wallis test, with Dunn’s post hoc test, has been used. Statistical analysis was performed using GraphPad Prism Version 6 (La Jolla, CA, USA).

## Results

A total of 406 patients had data collected from 14 individual acute hospital trusts covering the whole of the West Midlands region. The baseline and demographic details are shown in Table 1. The median age of patients was 56 years (range 16–91 years) and the majority of patients (78 %, *n* = 317) were undergoing elective surgery with the principal anesthetist being a consultant (74 %, *n* = 299) with junior anesthetists (with less than 4 years’ experience) accounting for only 8.5 % (*n* = 34) of cases. Volume control ventilation was the preferred mode of ventilation (67 %, *n* = 272) with the remaining patients (33 %, *n* = 134) being ventilated with a pressure-controlled modality.

**Table 1:** Baseline and demographic details of patients

Age, years	56 (40–70)
Gender, male *n* (%)	211 (52 %)
ASA (*n*)	
1	119 (29.3 %)
2	179 (44 %)
3	93 (22.9 %)
4	15 (3.7 %)
BMI	28 (24.6–32)
Class of surgery	
Elective	317 (78 %)
Expedited	15 (3.7 %)
Urgent	61 (15 %)
Emergency	8 (2 %)
Immediate	5 (1.2 %)
Specialty	
General surgery	117
ENT	76
Trauma and orthopedics	67
Urology	34
Gynecology	44
Vascular	21
Others	47
Duration	
Less than 1 h	130
1–2 h	157
2–4 h	81
Greater than 4 h	34
Tidal volume^a^ (ml)	500 (484–575)
pPeak^a^ (cmH_2_O)	20 (17–23)
PEEP^a^ (cmH_2_O)	4 (1–5)
FiO_2_^a^ (%)	50 (40–53)

Values are represented as total numbers with (%)

^a^The ventilation parameters collected and are median (IQR) values

**Fig 1:**
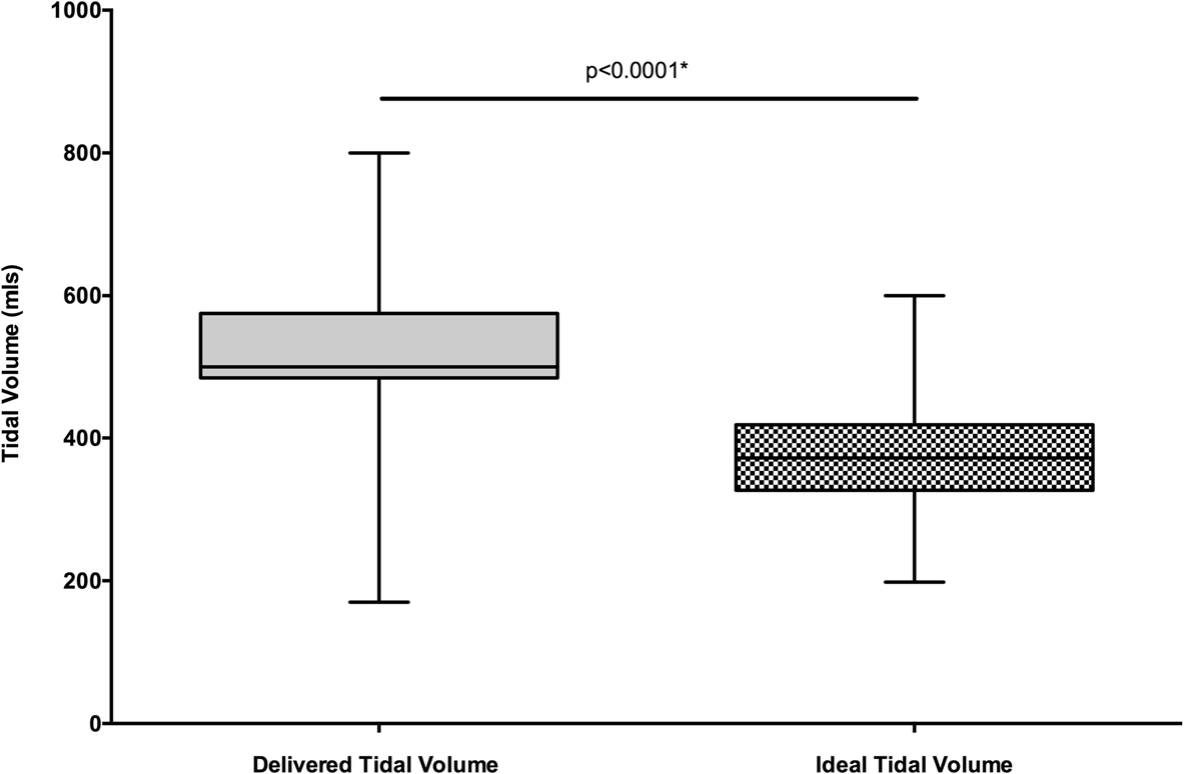
The difference between the actual delivered tidal volume and the ideal tidal volume based on predicted weight in the whole cohort of patients within the study. The *boxes* represent the median and IQR and the *whiskers* the minimum and maximum values. The *p* value is from a Wilcoxon signed rank test

Patients received a significantly greater median tidal volume compared to their ideal tidal volume (500 ml [IQR, 484–575 ml] vs. 372 ml [IQR, 327–418 ml] *p* < 0.001, Wilcoxon signed rank test; see Fig. 1). This equated to a median tidal volume of 8.4 ml/kg/PBW (IQR 7.5–9.7 ml/kg/PBW). A median of 4 cmH_2_O of PEEP (IQR 1–5 cmH_2_O) was used with no PEEP used in 37.4 % (*n* = 152) of cases. The median pPeak was 20 cmH_2_O (17–23 cmH_2_O), and the median FiO_2_ was 50 % (IQR 40–53 %).

A total of 116 patients met the criteria for major intra-abdominal surgery lasting greater than 2 h. Similar results were found in this subgroup analysis where patients did not receive lung-protective ventilation according to the modified IMPROVE trial definition used in this study. Again, these patients received a significantly greater median tidal volume compared to their ideal tidal volume based on PBW (525 ml [IQR 500–600 ml] vs. 375 ml [IQR 326–434 ml]; *p* < 0.001, Wilcoxon signed rank test) equivalent to 8.5 ml/kg/PBW (IQR 7.4–10.0 ml/kg/PBW). A PEEP of 4 cmH_2_O (IQR 3–5 cmH_2_O) was delivered with pPeak of 20 cmH_2_O (IQR 17–23 cmH_2_O) and a FiO_2_ of 50 % (IQR 44–55 %). Twenty-one (18 %) patients received no PEEP, and no patients received any recruitment maneuvers.

Patients undergoing urgent/emergency surgery were compared with those having elective surgery. The ventilator strategies were virtually identical with a median tidal volume of 500 ml, a median PEEP of 4 cmH_2_O, and a FiO_2_ of 50 %. Again, the tidal volumes delivered were significantly higher (*p* < 0.0001, Wilcoxon signed rank test) than those for their ideal body weight in both groups (see Table 2).

**Table 2:** Ventilator setting in patients depending upon urgency of surgery, ASA score, and BMI

	Actual TV^a^ (ml)	Ideal TV^a^ (ml)	pPeak (cmH_2_O)	PEEP (cmH_2_O)	FiO_2_ (%)
Elective (*n* = 317)	500 (500–570.8)	368 (326–413)	19 (17–22)	4 (0–5)	50 (40–53)
Emergency (*n* = 90)	500 (462–600)	386 (330–440)	21 (17–24)	4 (2–5)	50 (45–50.7)
ASA					
1–2 (*n* = 288)	500 (480–550)	369 (322–419)	19 (16–23)	4 (0–5)	50 (40–55)
3–4 (*n* = 99)	525 (500–600)	376 (340–376)	21 (18–24)	4 (2–5)	50 (43–55)
BMI (kg/m^2^)					
18.5–24.9 (*n* = 97)	500 (455–550)	379 (334–423)	17^b^ (14–20)	4 (1–5)	50 (45–53)
25–29.9 (*N* = 130)	500 (489–577)	369 (321–428)	19^b^ (16–22)	4 (0–5)	50 (40–55)
30–34.9 (*N* = 81)	500 (500–575)	360 (325–398)	21^b^ (18–24)	4 (0–5)	50 (40–52)
35–39.9 (*n* = 28)	545 (492–600)	389 (330–432)	23^b^ (20–26)	4 (0–5)	50 (40–52)
> 40 (*N* = 28)	500 (477–550)	356 (306–414)	24^b^ (22–27)	5 (2–5)	48 (40–50)

Values represent the median (IQR)

*ASA* American Society of Anesthesiology, *BMI* body mass index, *pPeak* peak pressure, *PEEP* positive end expiratory pressure, *FiO*_*2*_ fraction of inspired oxygen

^a^The difference between actual and the ideal tidal volumes (TV) was significant for all comparisons in all groups, *p* < 0.001 using a Wilcoxon signed rank test

^b^A Kruskal-Wallis test between the various BMI categories, where *p* < 0.0001. Dunn’s post hoc test demonstrated that the pPeak between each group was significantly different (*p* < 0.05) for each comparison

**Fig 2:**
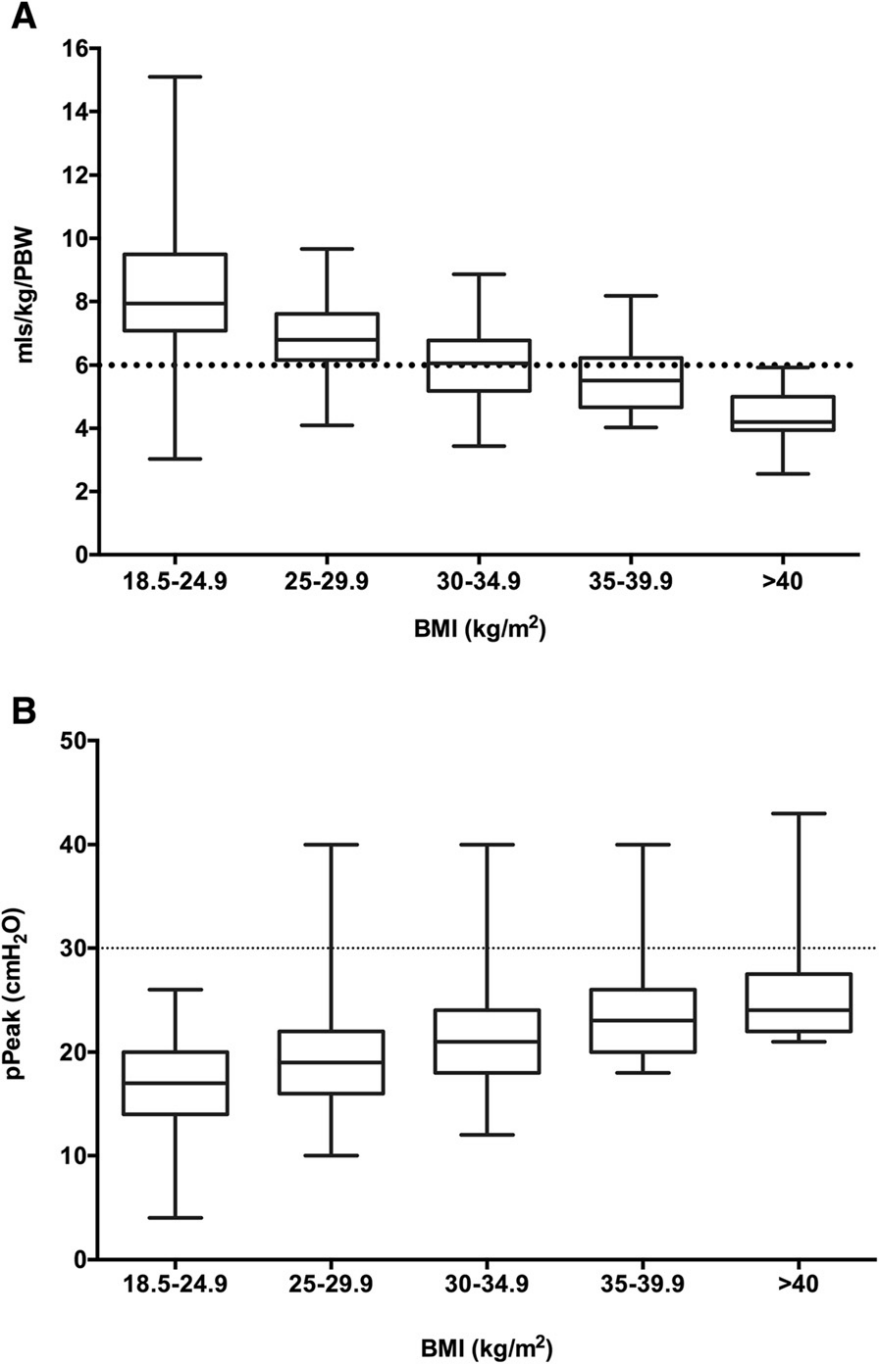
The tidal volume delivered based on actual weight (ml/kg) and peak pressure (pPeak) in patients categorized by their body mass index (BMI). The *boxes* represent the median and IQR and the *whiskers* the minimum and maximum values. **a** The tidal volumes (ml/kg). A Kruskal-Wallis test was significant (*p* < 0.001) with Dunn’s post hoc test demonstrating significant differences in between all comparisons. **b** The peak pressure (cmH_2_O). A Kruskal-Wallis test was significant (*p* < 0.001) with Dunn’s post hoc test demonstrating significant differences in between all comparisons

Patients were further divided by their body mass index (BMI) to identify if there was any relationship between weight/height and the ventilator strategy employed. Regardless of BMI, patients again received ventilation utilizing 500 ml TV with a FiO_2_ of 50 % and 4 cmH_2_O PEEP. As a consequence, patients with lower BMIs received higher tidal volumes compared with patients with greater BMIs (*p* < 0.001; Kruskal-Wallis); however, the pPeak delivered was lower in patients with lower BMIs (see Fig. 2).

## Discussion

There is increasing evidence emerging that the adoption of lung-protective ventilation in the perioperative period can reduce complications of major surgery within high-risk groups (Futier et al. 2013; Anaesthesiology PNIftCTNotESo et al. 2014; Futier et al. 2014; Ladha et al. 2015). This study has demonstrated that despite this, the adoption of these practices remains low amongst anesthetists within the West Midlands region of the UK. In fact, the study results suggest very little variability in the ventilation practices of anesthetists. Although a heterogeneous population undergoing mechanical ventilation was included, the subgroup analysis of the high-risk groups, based on ASA, BMI, urgency of surgery, and major abdominal surgery, suggested that even in these populations lung-protective ventilation has not been widely implemented.

The definition of lung-protective ventilation used was adopted from the largest RCT carried out in the perioperative setting and included low tidal volumes, a pPlateau of < 30 cmH_2_O, and a moderate use of PEEP (6–8 cmH_2_O). The routine use of recruitment maneuvers every 30 min as part of the bundle was excluded due to controversies over its effect (Futier et al. 2013). However, although no patients were ventilated according to these guidelines, the ventilation practices were comparable, with a tidal volume of 8.4 ml/kg/PBW being delivered and pPeak within the defined targets. The use of PEEP was poor with a large proportion of patients not receiving any PEEP. In those that did, PEEP levels were lower than suggested by Futier et al. These ventilator parameters are superior compared to those used in the “standard” group in the IMPROVE trial which received extremely large tidal volumes (10–12 ml/kg/PBW) and no PEEP, which are known to be harmful and as demonstrated in this cohort outdated as standard ventilation practice (Guldner et al. 2015; Ladha et al. 2015; Wanderer et al. 2015).

These results are similar to those reported by Levin et al. in 2014 in a retrospective study of approximately 29,000 patients in a single large center in the USA. They found that the median TV was 525 ml corresponding to 8.4 ml/kg/PBW, the median PEEP was 4, and the peak inspiratory pressure was 21 cmH_2_O. In their large cohort, they found that low tidal volumes (6 ml/kg/PBW) and the use of low levels of PEEP were associated with an increased risk of mortality, suggesting that merely reducing tidal volumes may not confer benefits and that an entire perioperative strategy may be required (Levin et al. 2014).

Due to the lack of variation in ventilator parameters set by anesthetists, patients with low BMIs received high tidal volumes, but presumably due to excellent chest wall and lung compliance, these higher tidal volumes resulted with conversely reduced levels of pPeak. The opposite effect of this was seen in patients with larger BMIs where lower tidal volumes (according to weight) were delivered but with significantly higher pPeak; however, it was rare for the pPeak to exceed 30 cmH_2_O (see Fig. 2).

The findings by Levin et al. and the study finding described raise the question as to how lung-protective ventilation should be defined and what is the principal driver of VILI? Is it barotrauma or volutrauma? The conventional theory from the ARDSNet trial was that lowering tidal volumes and adopting PEEP in ARDS resulted in improved mortality (Ventilation with Lower Tidal Volumes as Compared with Traditional Tidal Volumes for Acute Lung Injury and the Acute Respiratory Distress Syndrome, 2000; Levin et al. 2014). However, a recent publication by Amato et al. suggested that perhaps improving lung compliance with the judicious use of PEEP and recruitment maneuvers results in improved tidal volumes while at the same time limiting the driving pressure within the injured lung. They concluded that perhaps limitation of pressure was therefore more important than simply reduced tidal volume ventilation (Amato et al. 2015).

The majority of patients undergoing surgery have normal lungs, with preserved lung compliance leading many anesthetists to question the wisdom of translating findings from extremely sick patients with ARDS to those undergoing elective surgery, even if the surgery is considered major. However, the institution of general anesthesia and positive pressure ventilation are known to cause atelectasis in dependent areas of the lungs, which can interfere with postoperative oxygenation for several days afterwards. Additionally, atelectasis predisposes to the translocation and growth of bacteria, leading to pneumonia and subsequently ALI/ARDS (van Kaam et al. 2004; Sutherasan et al. 2014).

A recent large retrospective study of more than 65,000 patients undergoing surgery seemed to corroborate the finding of Amato et al. that pressure regulation is perhaps the key driver of VILI. The authors of this study suggested that lung protection should be employed for all surgical procedures regardless of risk and that in patients with no pre-existing lung disease the pPlateau should be as low as possible (less than 16 cmH_2_O), as in their cohort this was associated with a lower risk of pulmonary complications (Ladha et al. 2015).

This study did not investigate the perceived barriers to using lung-protective ventilation perioperatively. However, the use of low tidal volumes does reduce minute ventilation, necessitating an increase in respiratory rate. The use of PEEP by anesthetists remains low with potential reasons being the adverse effects on cardiovascular function and excessive venous congestion making some surgical procedures, such as liver surgery, technically more difficult (Anaesthesiology PNIftCTNotESo et al. 2014; Severgnini et al. 2013; Futier et al. 2014). Additionally, as perioperative hypoxia is relatively uncommon and the consequences of atelectasis are seen several days after, perhaps the benefits of PEEP and an “open-lung” strategy fail to be appreciated by all anesthetists (Levin et al. 2014). The publication of the PROVHILO trial comparing low PEEP (< 2 cmH_2_O) with a high PEEP (12 cmH_2_O) in patients undergoing major abdominal surgery may have added some degree of guidance as to the level of PEEP deemed optimal perioperatively, with no difference found between the groups for the incidence of pulmonary complications, with suggestions of harm in the high-PEEP group. However, a criticism of the trial was that the two groups were not truly reflective of current practice with PEEP setting at the extremes (very low vs. very high) and thus fails to guide practice over the appropriate levels of PEEP to use perioperatively (Anaesthesiology PNIftCTNotESo et al. 2014).

High levels of inspired oxygen (80 %) have been advocated for the prevention of surgical site infections; however, this may contribute to PPCs by increasing the tendency for absorption atelectasis and causing direct alveolar damage (Greif et al. 2000; Lumb & Walton 2012). At present, no consensus exists regarding the use of perioperative hyperoxia, however, within our results, the use of hyperoxia was moderate and correlated with the IMPROVE trial’s protocol of using inspired oxygen concentrations of 50 % (Kao et al. 2012; Togioka et al. 2012).

Our study had several limitations. Firstly, postoperative complications were not collected; hence, the findings cannot be correlated with patient-centered outcomes. Secondly, the data was sampled during a single snapshot of each operative case. Therefore, although the tidal volumes measured were likely to be reflective, as the vast majority of cases used a volume control mode, pPeak and PEEP may have altered and thus may not truly reflect overall care of patients. Thirdly, the definition used for lung-protective ventilation was based on a modified version of that employed in the IMPROVE trial. The IMPROVE trial only showed benefits of a lung-protective strategy in a high-risk population undergoing abdominal surgery. Hence, the use of such a strategy in all patients being ventilated for surgery has not been fully validated. Fourthly, the adequacy of ventilation was not assessed either by measurement of oxygen saturations, end-tidal carbon dioxide, or arterial blood gas analysis. Finally, as the data collection was not blinded, the delivery of ventilation by the principal anesthetist may have been altered to reflect a more lung-protective strategy.

## Conclusions

In conclusion, our study has demonstrated that anesthetists ventilate patients with low pressures and moderate tidal volumes, while the use of PEEP was low and recruitment maneuvers were not used. This strategy, although not fully compliant with a lung-protective strategy, does support evidence that lower tidal volumes are being employed in anesthesia and that the high tidal volume control groups (10–12 ml/kg) used in recent RCTs are not reflective of current practice (Futier et al. 2013). Additionally, this study suggests that ventilation strategies employed by anesthetists are not individualized to patients based on size, ASA score, or potential risk of PPC and that a “one-size-fits-all” approach remains.

How exactly to deliver lung protection perioperatively remains controversial, with additional trials, such as iPROVE (Ferrando et al. 2015), still being conducted, and as long as no consensus is reached, the adoption of these practices may remain low (Anaesthesiology PNIftCTNotESo et al. 2014; Futier et al. 2014; Ladha et al. 2015; Hartland et al. 2015). However, our study has highlighted that a strategy that employs low pressures and PEEP, at the very least, needs to be emphasized amongst anesthetists with particular focus on the benefits they may confer for patients’ outcomes.

## Abbreviations

ARDS: acute respiratory distress syndrome
BMI: body mass index
ICU: intensive care unit
IL-6: interleukin-6
IQR: inter-quartile range
PBW: predicted body weight
PEEP: positive end expiratory pressure
PPC: postoperative pulmonary complication
pPeak: peak pressure
RCTs: randomized controlled trials
SIRS: systemic inflammatory response syndrome
TNF-α: tumor necrosis factor-alpha
VILI: ventilator-induced lung injury

## References

[CR1] Amato MB, Barbas CS, Medeiros DM, Magaldi RB, Schettino GP, Lorenzi-Filho G (1998). Effect of a protective-ventilation strategy on mortality in the acute respiratory distress syndrome. N Engl J Med.

[CR2] Amato MB, Meade MO, Slutsky AS, Brochard L, Costa EL, Schoenfeld DA (2015). Driving pressure and survival in the acute respiratory distress syndrome. N Engl J Med.

[CR3] Hemmes SN, Gama De A, Gama De Abreu M, Pelosi P, Schultz MJ, Anaesthesiology PNIftCTNotESo (2014). High versus low positive end-expiratory pressure during general anaesthesia for open abdominal surgery (PROVHILO trial): a multicentre randomised controlled trial. Lancet.

[CR4] Barbas CS, de Mattos GF, Borges ER (2005). Recruitment maneuvers and positive end-expiratory pressure/tidal ventilation titration in acute lung injury/acute respiratory distress syndrome: translating experimental results to clinical practice. Crit Care.

[CR5] Ferrando C, Soro M, Canet J, Unzueta MC, Suarez F, Librero J (2015). Rationale and study design for an individualized perioperative open lung ventilatory strategy (iPROVE): study protocol for a randomized controlled trial. Trials.

[CR6] Fisher BW, Majumdar SR, McAlister FA (2002). Predicting pulmonary complications after nonthoracic surgery: a systematic review of blinded studies. Am J Med.

[CR7] Futier E, Constantin JM, Paugam-Burtz C, Pascal J, Eurin M, Neuschwander A (2013). A trial of intraoperative low-tidal-volume ventilation in abdominal surgery. N Engl J Med.

[CR8] Futier E, Constantin JM, Jaber S (2014). Protective lung ventilation in operating room: a systematic review. Minerva Anestesiol.

[CR9] Greif R, Akca O, Horn EP, Kurz A, Sessler DI, Outcomes RG (2000). Supplemental perioperative oxygen to reduce the incidence of surgical-wound infection. N Engl J Med.

[CR10] Guldner A, Kiss T, Serpa Neto A, Hemmes SN, Canet J, Spieth PM (2015). Intraoperative protective mechanical ventilation for prevention of postoperative pulmonary complications: a comprehensive review of the role of tidal volume, positive end-expiratory pressure, and lung recruitment maneuvers. Anesthesiology.

[CR11] Hartland BL, Newell TJ, Damico N (2015). Alveolar recruitment maneuvers under general anesthesia: a systematic review of the literature. Respir Care.

[CR12] Imai Y, Parodo J, Kajikawa O, de Perrot M, Fischer S, Edwards V (2003). Injurious mechanical ventilation and end-organ epithelial cell apoptosis and organ dysfunction in an experimental model of acute respiratory distress syndrome. JAMA.

[CR13] Kao LS, Millas SG, Pedroza C, Tyson JE, Lally KP (2012). Should perioperative supplemental oxygen be routinely recommended for surgery patients? A Bayesian meta-analysis. Ann Surg.

[CR14] Ladha K, Vidal Melo MF, McLean DJ, Wanderer JP, Grabitz SD, Kurth T (2015). Intraoperative protective mechanical ventilation and risk of postoperative respiratory complications: hospital based registry study. BMJ.

[CR15] Lellouche F, Dionne S, Simard S, Bussieres J, Dagenais F (2012). High tidal volumes in mechanically ventilated patients increase organ dysfunction after cardiac surgery. Anesthesiology.

[CR16] Levin MA, McCormick PJ, Lin HM, Hosseinian L, Fischer GW (2014). Low intraoperative tidal volume ventilation with minimal PEEP is associated with increased mortality. Br J Anaesth.

[CR17] Lim LH, Wagner EM (2003). Airway distension promotes leukocyte recruitment in rat tracheal circulation. Am J Respir Crit Care Med.

[CR18] Lumb AB, Walton LJ (2012). Perioperative oxygen toxicity. Anesthesiol Clin.

[CR19] Minkovich L, Djaiani G, Katznelson R, Day F, Fedorko L, Tan J (2007). Effects of alveolar recruitment on arterial oxygenation in patients after cardiac surgery: a prospective, randomized, controlled clinical trial. J Cardiothorac Vasc Anesth.

[CR20] Nielsen J, Ostergaard M, Kjaergaard J, Tingleff J, Berthelsen PG, Nygard E (2005). Lung recruitment maneuver depresses central hemodynamics in patients following cardiac surgery. Intensive Care Med.

[CR21] Sakr Y, Vincent JL, Reinhart K, Groeneveld J, Michalopoulos A, Sprung CL (2005). High tidal volume and positive fluid balance are associated with worse outcome in acute lung injury. Chest.

[CR22] Serejo LG, da Silva-Junior FP, Bastos JP, de Bruin GS, Mota RM, de Bruin PF (2007). Risk factors for pulmonary complications after emergency abdominal surgery. Respir Med.

[CR23] Severgnini P, Selmo G, Lanza C, Chiesa A, Frigerio A, Bacuzzi A (2013). Protective mechanical ventilation during general anesthesia for open abdominal surgery improves postoperative pulmonary function. Anesthesiology.

[CR24] Slutsky AS, Ranieri VM (2013). Ventilator-induced lung injury. N Engl J Med.

[CR25] Sutherasan Y, Vargas M, Pelosi P (2014). Protective mechanical ventilation in the non-injured lung: review and meta-analysis. Crit Care.

[CR26] Tao T, Bo L, Chen F, Xie Q, Zou Y, Hu B (2014). Effect of protective ventilation on postoperative pulmonary complications in patients undergoing general anaesthesia: a meta-analysis of randomised controlled trials. BMJ open.

[CR27] Togioka B, Galvagno S, Sumida S, Murphy J, Ouanes JP, Wu C (2012). The role of perioperative high inspired oxygen therapy in reducing surgical site infection: a meta-analysis. Anesth Analg.

[CR28] Tremblay LN, Miatto D, Hamid Q, Govindarajan A, Slutsky AS (2002). Injurious ventilation induces widespread pulmonary epithelial expression of tumor necrosis factor-alpha and interleukin-6 messenger RNA. Crit Care Med.

[CR29] van Kaam AH, Lachmann RA, Herting E, De Jaegere A, van Iwaarden F, Noorduyn LA (2004). Reducing atelectasis attenuates bacterial growth and translocation in experimental pneumonia. Am J Respir Crit Care Med.

[CR30] Ventilation with lower tidal volumes as compared with traditional tidal volumes for acute lung injury and the acute respiratory distress syndrome. New England Journal of Medicine. 2000;342(18):1301–8. 10.1056/NEJM200005043421801 10793162

[CR31] Wanderer JP, Ehrenfeld JM, Epstein RH, Kor DJ, Bartz RR, Fernandez-Bustamante A (2015). Temporal trends and current practice patterns for intraoperative ventilation at U.S. academic medical centers: a retrospective study. BMC Anesthesio.

[CR32] Wrigge H, Uhlig U, Zinserling J, Behrends-Callsen E, Ottersbach G, Fischer M, et al. The effects of different ventilatory settings on pulmonary and systemic inflammatory responses during major surgery. Anesth Analg. 2004;98:775–81. table of contents. 10.1213/01.ane.0000100663.11852.bf 14980936

